# Helical orbitals and circular currents in linear carbon wires†
†Electronic supplementary information (ESI) available: Orbital splitting and zeroth-order Green's function; substituent effects in 1,5-disubstituted [4]cumulenes; substituent effect of pyramidalized single-faced π-donors; aryl-substituted cumulene; barriers between conformations; [5]cumulene transmission; coordinate transformation and current density convergence; wide-band transmission plots. See DOI: 10.1039/c8sc05464a


**DOI:** 10.1039/c8sc05464a

**Published:** 2019-03-19

**Authors:** Marc H. Garner, Anders Jensen, Louise O. H. Hyllested, Gemma C. Solomon

**Affiliations:** a Department of Chemistry , Nano-Science Center , University of Copenhagen , Universitetsparken 5 , DK-2100 , Copenhagen Ø , Denmark . Email: marc@chem.ku.dk ; Email: gsolomon@chem.ku.dk

## Abstract

Disubstituted odd-carbon cumulenes are linear carbon wires with near-degenerate helical π-orbitals. Such cumulenes are chiral molecules but their electronic structure consists of helical orbitals of both chiralities. For these helical molecular orbitals to give rise to experimentally observable effects, the near-degenerate orbitals of opposite helicities must be split. Here we show how pyramidalized single-faced π-donors, such as the amine substituent, provide a strategy for splitting the helical molecular orbitals. The chirality induced by the amine substituents allow for systematic control of the helicity of the frontier orbitals. We examine how the helical orbitals in odd-carbon cumulenes control the coherent electron transport properties, and we explicitly predict two modes in the experimental single-molecule conductance for these molecules. We also show that the current density through these linear wires exhibits strong circular currents. The direction of the circular currents is systematically controlled by the helicity of the frontier molecular orbitals, and is therefore altered by changing between the conformations of the molecule. Furthermore, the circular currents are subject to a full ring-reversal around antiresonances in the Landauer transmission, emphasizing the relation to destructive quantum interference. With circular currents present around truly linear carbon wires, cumulenes are promising candidates for novel applications in molecular electronics.

## Abstract 2

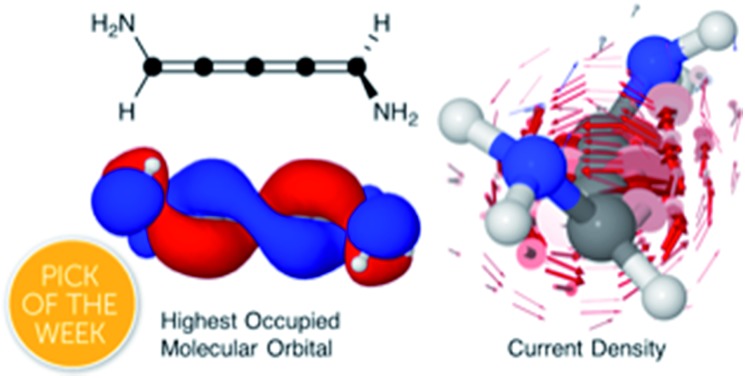
Disubstituted odd-carbon cumulenes are linear carbon wires with helical π-orbitals, which results in circular current around the wire.

## Introduction

Cumulenes are a series of linear carbon wires similar to polyynes but terminated by tricoordinate carbon atoms. By convention, we label cumulenes by the number of double bonds *n*, and the number of cumulenic carbon atoms is thus *n* + 1. We refer to the even *n* series simply as even [*n*]cumulenes, where the shortest member (*n* = 2) is best known as allene. The two orthogonal π-orbital systems of cumulenes are separated by symmetry. Thus, the odd [*n*]cumulenes have mutually planar end-groups, while the even [*n*]cumulenes have mutually perpendicular end-groups in order to form closed-shell systems.

**Fig. 1:**
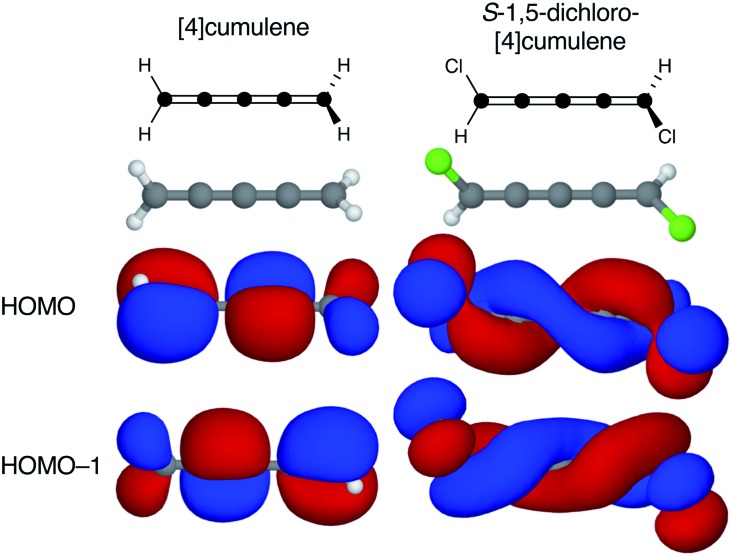
Optimized molecular structure and the two highest occupied molecular orbitals of [4]cumulene and *S*-1,5-dichloro-[4]cumulene.

Shown in Fig. 1 from a density functional theory (DFT) calculation, the even [*n*]cumulenes have the unique property that a reduction of symmetry from *D*_2d_ to *C*_2_ by an α,ω-disubstitution causes the otherwise orthogonal π-orbitals to mix and form a new set of helical molecular orbitals. This intriguing property of even [*n*]cumulenes was coined electrohelicity by Hendon *et al.*,^1^ and we recently described this phenomenon in detail.^2^ For each pair of degenerate π-orbitals in the unsubstituted cumulene (Fig. 1, left) a corresponding pair of quasi-degenerate helical orbitals appear in the disubstituted case (Fig. 1, right). While the substituted molecule is one of two enantiomers, here the *S*-enantiomer, both chiralities are present in the electronic structure. The highest occupied molecular orbital (HOMO) of *S*-1,5-dichloro-[4]cumulene is a *P*-helix and the HOMO–1 is an *M*-helix; this order is reversed for the *R*-enantiomer as inferred by parity. The appearance of helical orbitals is a consequence of the helicogenic C_2_ rotation symmetry of even [*n*]cumulenes, which is not present in the ground-state geometries of odd [*n*]cumulenes and polyynes.^2^ As we presented in recent work, the electronic structure of even [*n*]cumulenes is best understood by considering them as coarctate Möbius systems.^3^,^4^ The helical molecular orbitals are an intrinsic property of such systems when helicogenic symmetry is present, and they can be derived using a simple Hückel model and group theory.^2^

Beautiful and conceptually interesting as the helical molecular orbitals are, they are not experimental observables. It is essential to explore what novel effects may arise from helical orbitals, and here we consider how such effects may be used in molecular electronics. We envision that it will be possible to incorporate electronic functionality into otherwise linear atomic carbon wires,^5^–^8^ given frontier molecular orbitals of both helicities can be present.

Helical orbitals appear in electronic structure calculations on carbon chains whenever appropriate helicogenic symmetry is achieved,^1^,^2^,^9^–^14^ for example through mechanical torsion along the molecular axis.^15^–^18^ While axial torsion may be a promising avenue, only the even [*n*]cumulene series has the appropriate molecular symmetry in the ground-state structure. Though cumulenes are not as stable as polyynes, the even [*n*]cumulenes have been synthesized up to the [6]cumulene,^19^–^25^ the odd [*n*]cumulenes up to [9]cumulene,^26^ and metallacumulenes of similar lengths have been reported.^27^,^28^ Functionalized chiral allenes have been characterized,^29^–^31^ and in recent work Ozcelik *et al.*^32^ presented conductance measurements on a monolayer of enantiopure allenes. Still, it is clear that strategies for further stabilization of linear carbon wires must be conceived for them to be utilized in single-molecule devices; rotaxane-protected cumulene and polyyne wires were recently reported by Tykwinski and co-workers and hold promise in this regard.^33^–^35^

Here we present a theoretical demonstration of the utilization of even [*n*]cumulenes and their helical orbitals in single-molecule conductance calculations. First, we explore substituent strategies for tuning the order and the energetic splitting of the otherwise near-degenerate frontier molecular orbitals. Second, we show how the splitting of orbitals of opposite helicity controls the electronic transmission through single-molecule junctions, which allows us to make explicit predictions for the single-molecule conductance. Finally, we examine the low-bias current density of these junctions and reveal strong circular currents around the linear wires with the direction of the currents being controlled explicitly by the helicity of the frontier orbitals.

## Methods

All electronic structure calculations presented in the manuscript are performed using DFT as implemented in the Atomic Simulation Environment (ASE) and GPAW.^36^–^38^ The Perdew–Burke–Ernzerhof (PBE) exchange-correlation functional is applied with double-ζ plus polarization basis set for all atoms.^39^ This method is chosen for the purpose of electron transport calculations, and we compare the vacuum results with calculations using the M06-2X functional and Hartree–Fock method in ESI.† All molecules have been optimized in vacuum to a force threshold of 5 meV Å^–1^. Molecular structures and orbitals are plotted using Jmol with the default isosurface cutoff value 0.02.^40^

### Electron transport calculations

We employ the non-equilibrium Green's functions (NEGF) approach as implemented in GPAW.^41^ Single-molecule junctions are constructed by placing the molecule between two four-atom Au pyramids on 4 × 4 Au(111) surfaces with periodic boundary conditions in the plane of the surfaces. The junction structure is relaxed to a force threshold of 0.05 eV Å^–1^ with all Au atoms kept fixed during the optimization. From this junction structure the Landauer transmission *T*(*E*) is calculated using eqn (1) with a *k*-point sampling over a 4 × 4 × 1 Monkhorst–Pack mesh in the first Brillouin zone,

1 *T*(*E*) = tr[*Γ*_L_(*E*)*G*^r^(*E*)*Γ*_R_(*E*)*G*^a^(*E*)]

where *Γ*_L/R_ are the coupling matrices of the left and right electrodes. The junction structure constitutes the extended molecular region and with its Hamiltonian *H*_M_ and overlap matrix *S*_M_ and the calculated self-energies of the left and right electrodes *Σ*_L/R_, the advanced and retarded Green functions *G*^r/a^ are calculated using eqn (2),

2 *G*^r/a^(*E*) = [*E*·*S*_M_ – *H*_M_ – *Σ*r/aL(*E*) – *Σ*r/aR(*E*)]^–1^.

### Current density calculations

To calculate the low-bias current density through a single-molecule junction, we use a simpler junction setup due to computational constraints. We employ the NEGF approach as described above (eqn (1) and (2)) using GPAW. Our approach is described in full detail elsewhere,^42^ and the code is available online at ; https://github.com/marchgarner/Current_Density.git; a brief description follows here.

Single-molecule junctions are constructed with the molecule placed between two s-band electrodes, which are formed using two dihydrogen molecules with a N–H distance of 1.5 Å, thus forming the extended molecular region used in eqn (2). A single-ζ basis set is used for the hydrogens, and the s-band electrodes are formed using the wide-band approximation with nonzero matrix elements of *Γ*_L/R_ set to *γ* = 1.0 eV. As we have recently reported for a large set of molecules,^43^ this approach to calculating the transmission gives a result that is qualitatively similar to that using periodic three-dimensional Au electrodes; this is also evident for cumulenes based on Fig. S5, S9, and S10 in ESI.†

In the absence of an external magnetic field and at zero-temperature, the low-bias current density **j**(**r**) can be calculated within the NEGF formalism as
3

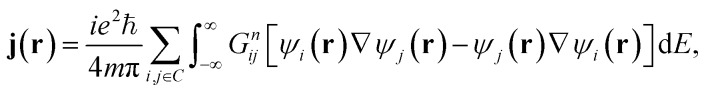

where *ψ* are basis functions, and *C* are the functions within the extended molecular region which we sum over.^44^,^45^ *G*^*n*^ is the non-equilibrium part of the lesser Green's function

4 *G*^*n*^ = *iG*^r^*Γ*_L_*G*^a^δ*V*,

with δ*V* being a small symmetric bias difference between the electrodes, which we set to δ*V* = 1 mV. The total current *J* can be calculated by integrating the current density over a cross-section area
5

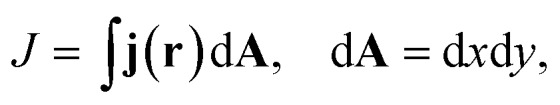


*z* being the direction of the current. The total current *J* should be constant throughout the junction, but as a finite basis set is used the current will often not fully converge to the total current which can be calculated with the standard Landauer–Büttiker formula. As we show in ESI Part VII,† such deviation can be particularly large close to the electrodes but is less significant along the linear carbon wire. Therefore, we only plot the current density field from the first to the last carbon atom.

**Fig. 2:**
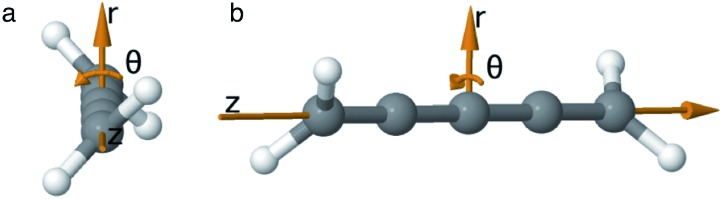
Orientation of [4]cumulene in the cylindrical coordinate system shown from end-view (a) and side-view (b).

As we want to explore the relation between the current density and helical molecular orbitals, it is instructive to analyse the vector field in a cylindrical coordinate system. The conversion from Cartesian coordinates is trivial and is included for reference in ESI Part VII.† As shown in Fig. 2, we align the carbon axis with the *z*-axis thereby placing it in the origin of the *r* and *θ* dimensions. The colour of each arrow is scaled by the *z* or *θ* vector-component normalized by the vector length (the Euclidean norm), thereby giving it a value between –1 and +1.
6

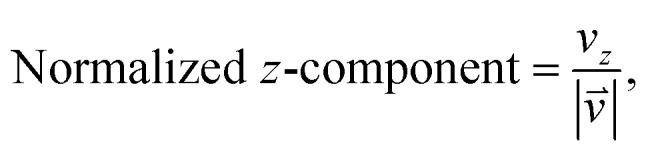



7

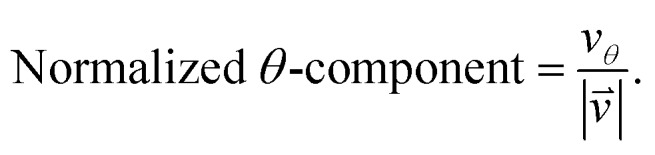




Each vector in the current density field is plotted as an arrow of same length. The length of the vector (the magnitude of the current density at position **r**) is the radius of the arrow, which is scaled relative to the largest arrow in each plot. Vectors smaller than 5% of the largest *z*-component (*v_z_*) are not shown.

## Splitting helical molecular orbitals

By adding two substituents to an even [*n*]cumulene its frontier molecular orbitals become helical as the symmetry is reduced from *D*_2d_ to *C*_2_ (see Fig. 1 and ref. 2). In the *C*_2_ point group there are no degenerate irreducible representations and consequently the HOMO and HOMO–1 are explicitly non-degenerate in the disubstituted molecule. However, every pair of helical π-orbitals remain quasi-degenerate; in the case of *S*-1,5-dichloro-[4]cumulene the energy difference between the HOMO and HOMO–1 is only 6 meV. The energetic splitting between the near-degenerate pairs of helical orbitals is important for several reasons.

First, for the helical orbitals to result in some observable of interest in an experiment, the energetic splitting must be large so the orbitals of opposite helicity do not change order due to structural fluctuations.

Second, the helical orbitals are more than just helical. In the broad sense, they are fully delocalized molecular orbitals spanning the full length of the molecule. The rectilinear π-orbitals of the unsubstituted [4]cumulene (Fig. 1a) do not extend from the p-orbital on first carbon atom onto the p-orbital of the last carbon atom, the helical π-orbitals of the substituted molecule do (Fig. 1b). In this sense the energetic splitting of the frontier molecular orbitals is a measure of the deviation from *D*_2d_ symmetry: from localized π-orbitals to delocalized helical orbitals.

Finally, the energetic splitting between a pair of degenerate molecular orbitals is essential for the coherent electron transport properties, which is the main focus of this study. Within the approximations of the Hückel method, the contribution to the total transmission from each pair of near-degenerate molecular orbitals of opposite symmetry is proportional to the square of the splitting of the two orbitals as shown in ESI Part I.† 46,47 Thus, from the simplest orbital model, the contribution to the electronic transmission from a pair of near-degenerate helical orbitals is predicted to be negligible. As we shall demonstrate, this is a consequence of destructive quantum interference.^48^–^50^

### Splitting helical orbitals with substituents

Any pair of substituents that preserves the helicogenic *C*_2_ symmetry of the molecule can be used to reduce the symmetry and obtain helical molecular orbitals. However, simple substituents have little effect on the energetic splitting of the helical orbitals. As we show in ESI Part II,† the electron withdrawing or accepting character of the substituents has only marginal influence on the splitting of the near-degenerate orbital pairs. Furthermore, the order of the helical orbitals is not controlled by the choice of substituent, thus for any *S*-enantiomer we find the HOMO to be a *P*-helix and the HOMO–1 to be an *M*-helix. In other words, electron withdrawing and accepting substituents are not found to produce an opposite effect on the helical orbitals.

### Pyramidalized single-faced π-donors

**Fig. 3:**
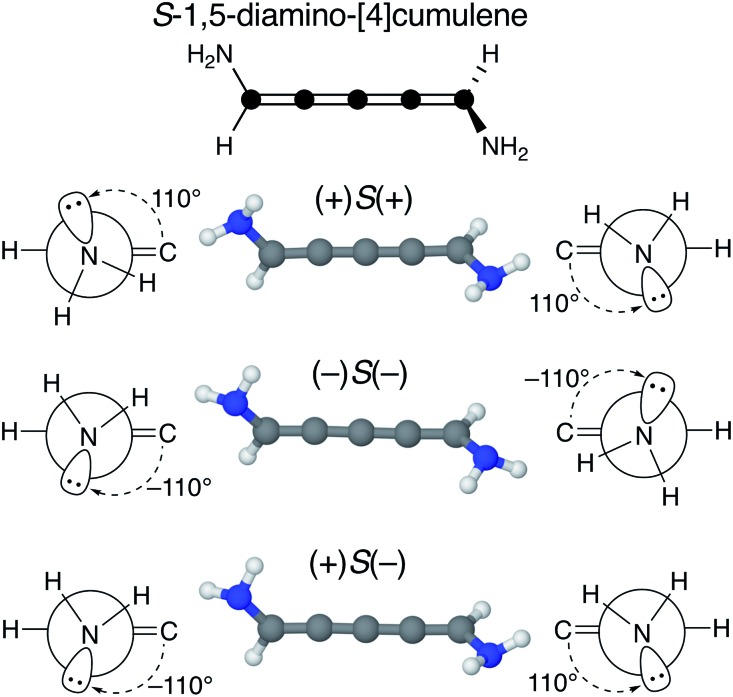
Optimized molecular structure and Newman projections along each C–N axis (approximate). The three distinguishable conformations of *S*-1,5-diamino-[4]cumulene are shown. The ± denotes the plus or minus configuration of the pseudo dihedral angle (approximately ±110°) measured at C=C–N–lone-pair, and the *S*/*R* denotes the axial chirality of the molecule.

We find that substituents that provide additional stereogenic centres while retaining the *C*_2_ symmetry of the molecule can provide both significant energetic splitting and tune the order of the helical frontier orbitals. Pyramidalized single-faced π-donors are a class of such substituents commonly used in organic chemistry, the simplest being the amine (–NH_2_) substituent. In Fig. 3, the three optimized conformations of *S*-1,5-diamino-[4]cumulene are shown. The two hydrogen atoms on each amine bend, which is generally perceived as the consequence of the nitrogen having a lone-pair orbital. Given the axial chirality of the molecule (*S* in this case), each nitrogen lone-pair will point in one of two directions which is described by the (approximate) C=C–N–lone-pair dihedral angle. As we find by optimization of the conformations, this dihedral angle is approximately ±110° as depicted by Newman projections in Fig. 3. The three distinguishable combinations of the lone-pairs give rise to the three conformations, which we name by whether each lone-pair is in the + or – configuration, *e.g.* (+)*S*(+) is the *S*-enantiomer with both lone-pairs in the +110° configuration. This is just a designation of the absolute chirality of the three conformations, which are related by diastereomerism. Each of these three have a corresponding mirror-image, for example, that of (+)*S*(+) will be (–)*R*(–).

**Fig. 4:**
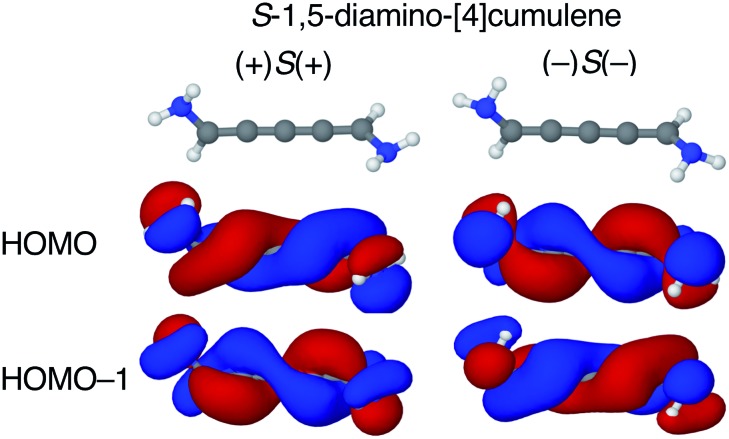
HOMO and HOMO–1 of (+)*S*(+) and (–)*S*(–) conformations S-1,5-diamino-[4]cumulene. The HOMO of (+)*S*(+) is an *M*-helix and the HOMO–1 is a *P*-helix; the order is reversed for the (–)*S*(–) conformation.

The configuration of the amine lone-pairs, each of them a chiral centre, affects the order of the helical molecular orbitals. Shown in Fig. 4, in the (+)*S*(+) conformation of *S*-1,5-diamino-[4]cumulene the HOMO is an *M*-helix and the HOMO–1 a *P*-helix. This order is reversed in the (–)*S*(–) conformation where the HOMO is a *P*-helix and the HOMO–1 an *M*-helix. The frontier molecular orbitals of these two conformations look like mirror images as the helicity of each orbital is opposite; but these two conformations are not each other's mirror images as both are *S*-enantiomers with regard to the axial chirality.

The energy splitting is also notably stronger with pyramidalized single-faced π-donors. Listed in Table 1, splitting of the HOMO and HOMO–1 are over an order of magnitude larger for the amine-substituted [4]cumulene than for the chloro-substituted case. Shown in Fig. S1 in ESI,† the frontier orbitals of the (+)*S*(–) conformation lose most of their helicity as the orbitals are near-degenerate (splitting is similar to the chloro-substituted case, see Table 1) and the symmetry is reduced to *C*_1_. The fact that the HOMO and HOMO–1 remain quasi-degenerate in the (+)*S*(–) conformation confirms that it is the lone-pair configurations that control the orbital splitting; having two lone-pairs of opposite chirality thus leads to a cancellation of the splitting effect on the helical orbitals.

**Table 1:** Energy splitting of the HOMO and HOMO–1 of 1,5-disubstituted [4]cumulenes^*a*^

NH_2_ (+)*S*(+)	NH_2_ (–)*S*(–)	NH_2_ (+)*S*(–)	Cl
78	76	5	6

^*a*^Given as absolute energy difference in meV.

The amine substituent is not a special case, and similar substituents such as dimethylamine and phosphine provide even larger splitting in the frontier orbitals of [4]cumulene, as shown in ESI Part III.† It appears that any substituent with a tilted orbital system (making it an additional stereogenic centre in an even [*n*]cumulene) can be used for systematically splitting and tuning the order of helical molecular orbitals. A range of realistic substituted cumulenes are likely to exist where this end-group effect on the helical π-system can be utilized.^51^ For example, aryl-substituted cumulenes have this property as we demonstrate in ESI Part IV.† Such systems have been reported in the literature and may hold promise for further functionalization at the chemically stable phenyl rings.^52^–^56^

The possibility of systematically splitting the helical frontier orbitals of cumulenes by chemical design opens the possibility of experimental verification of the existence of helical orbitals spanning the length of linear molecules; a verification which ideally should be found by revisiting spectroscopic experiments on optical activity^11^,^57^–^59^ or with novel orbital imaging techniques.^60^–^66^ Here, we will explore how the helical orbitals influence the coherent electron transport properties of even [*n*]cumulenes with the aim of making explicit predictions for single-molecule conductance experiments.

## Electron transport properties

**Fig. 5:**
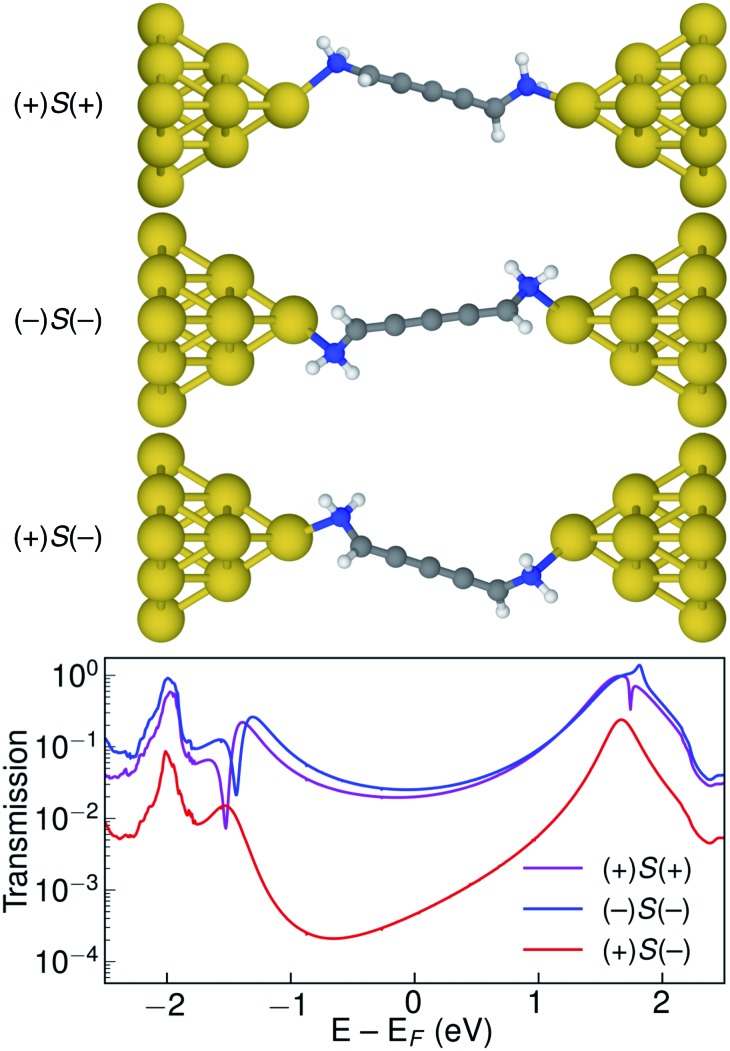
Optimized junction structures for (+)*S*(+), (–)*S*(–), and (+)*S*(–) conformations of 1,5-diamino-[4]cumulene and transmissions plotted semilogarithmically against energy. Only tip Au atoms are shown for clarity.

Let us now consider the coherent electron transport properties of the three conformations of *S*-1,5-diamino-[4]cumulene. Amines are common binding groups in single-molecule junctions because the nitrogen lone-pair forms a donor–acceptor bond to the gold electrode.^67^ Therefore three junction geometries can be made, as shown in Fig. 5, which have a one-to-one correspondence with the three conformations of the free molecule.

In the bottom panel of Fig. 5, the calculated Landauer transmission is shown for the three Au–molecule–Au junctions. In a broad energy range around the Fermi energy the (+)*S*(+) and (–)*S*(–) conformations have high transmission, while the transmission of the (+)*S*(–) conformation is almost two orders of magnitude lower as the HOMO and HOMO–1 remain quasi-degenerate (see Table 1). The phases of each molecular orbital differ at one of the nitrogen atoms (Fig. 4 and S1†), and consequently the transmission is suppressed due to orbital symmetry unless the near-degeneracy is broken.^46^,^47^ The high transmission of the (+)*S*(+) and (–)*S*(–) conformations is in direct agreement with the prediction that the energy splitting of the otherwise degenerate frontier orbital pairs control the transmission at the Fermi energy as we show in ESI Part I,† and originally demonstrated by Yoshizawa and co-workers.^46^,^47^

While the three junction structures shown in Fig. 5 are very similar, the transmissions of the three clearly differ and are a testament to the special electronic structure of even [*n*]cumulenes. As described in previous work and in ESI Part V,† no such behaviour is seen for the equivalent conformations of odd [*n*]cumulenes.^68^,^69^ The apparent transmission-suppression in even [*n*]cumulenes agrees with recently proposed guidelines for destructive quantum interference in the isolobal Möbius annulenes.^2^,^70^,^71^ Although substituents have been demonstrated to effectively tune conductance and specifically interference effects,^72^–^75^ it is unusual that the anchoring groups directly impact destructive quantum interference.^76^,^77^ Unlike anchoring groups in general, the chiral amine substituents provide a direct effect on the helical electronic structure of even [*n*]cumulenes and thus control their electronic transmission.

An explicit prediction for single-molecule junction experiments emerges from these results. For a racemic mixture of 1,5-diamino-[4]cumulene, or an equivalently substituted system, the high and low transmission modes will result in two separate peaks in a single-molecule conductance histogram. This prediction does not depend on the separation of the different conformations and enantiomers, it only requires that each measurement can be done on a timescale faster than that of the interconversion between conformations. For the case of the amine-substituted [4]cumulene, such an interconversion will be rapid for the free molecule. However, in ESI Part VI† we estimate the barrier for the bound molecule to ∼0.3 eV (∼7 kcal mol^–1^). While the timescale of interconversion will depend on the specific choice of substituent and experimental environment, the interconversion barrier is approaching a magnitude where the bimodal conductance can be resolved in break-junction experiments at room temperature.

## Current density through cumulenes

In the low-bias regime, electron transport through a single-molecule junction proceeds *via* a tunnelling mechanism through broadened molecular orbitals that are weakly coupled to the electrode states.^67^ As implied in eqn (3) and (4), the current density through a molecular device is intimately linked to its electronic structure.^42^,^78^–^80^ As we examine the relation between structure and current density in cumulenes, we note that the s-band electrodes are depicted as dihydrogen molecules, and that we systematically utilize colour-schemes to show the direction of each vector as described by eqn (6) and (7) in the Methods section.

### Current through rectilinear π-orbitals

**Fig. 6:**
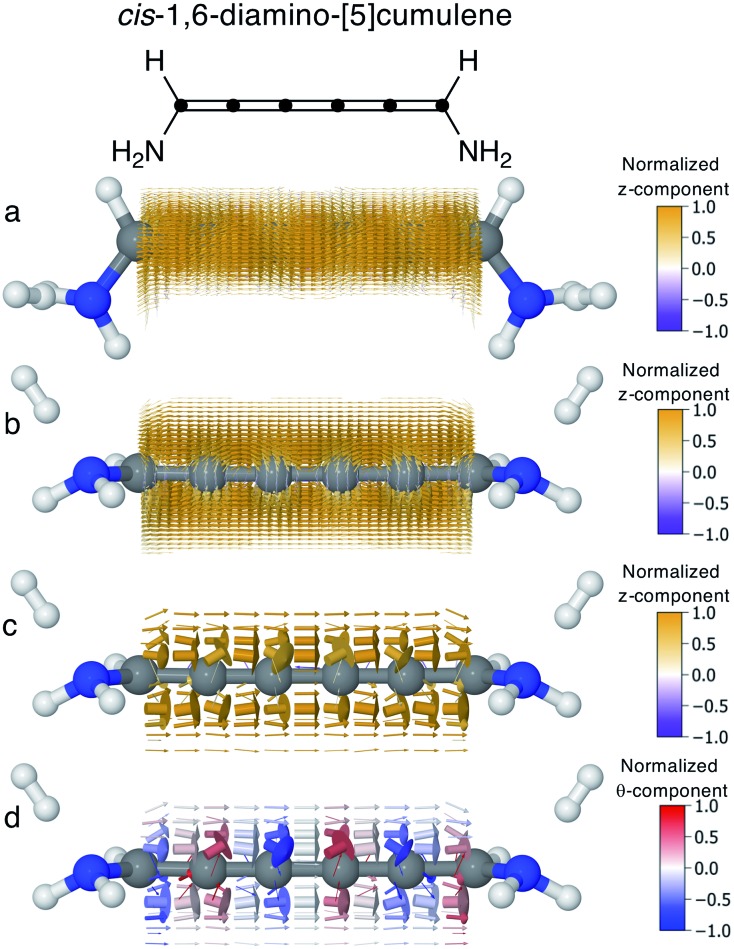
Current density calculated at the Fermi energy for *cis*-1,6-diamino-[5]cumulene; the 6-carbon cumulene with normal rectilinear π-orbitals. (a) Vector field calculated on a high-density grid and coloured by the *z*-component shown from top-view. (b) Vector field calculated on a high-density grid and coloured by the *z*-component shown from side-view, with the nodal plane in the current clearly visible. (c) Vector field calculated on a low-density grid and coloured by the *z*-component shown from side-view. (d) Vector field calculated on a low-density grid and coloured by the *θ*-component shown from side-view.

In conjugated molecules the π nodal plane is normally preserved in the current density, and as shown in Fig. 6 the odd [*n*]cumulenes are no different. By plotting the current density vector field in different ways in Fig. 6 we highlight different characteristic traits. By plotting it on a dense spatial grid in Fig. 6a and b it is clear that the vector field is a continuous function that is dominated by linear currents with a nodal plane throughout the molecule. By magnifying the arrows on a coarser grid in Fig. 6c, we can appreciate the direction of each arrow and, as highlighted by the colour scheme, most currents are in the direction of the total current. Changing the colour-scheme of the vector field does not change these conclusions. Shown in Fig. 6d, the vector field is plotted on a grid identical to Fig. 6c but instead coloured by the normalized *θ*-component (see eqn (7)). While there are fluctuations in the current density giving rise to non-zero *θ*-components, the current density is predominantly linear through the molecule.

### Current through helical π-orbitals

**Fig. 7:**
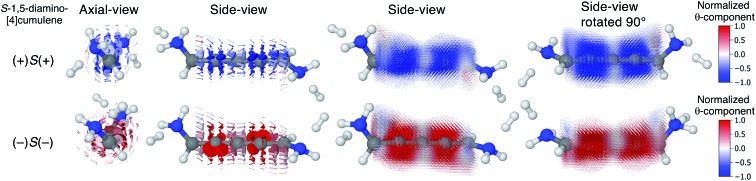
Current density calculated at the Fermi energy for the (+)*S*(+) and (–)*S*(–) conformations of 1,5-diamino-[4]cumulene. The vector field is shown on both low and high-density grids and is colored by the *θ*-component.

In even [*n*]cumulenes the frontier molecular orbitals that carry the current are helical; in the case of 1,5-diamino-[4]cumulene the helicity of the frontier orbitals is systematically controlled by the conformation of the molecule. In Fig. 7 the current densities of the (+)*S*(+) and (–)*S*(–) conformations are shown coloured by the *θ*-component. Both conformations exhibit clear circular currents around the linear wire, particular around the second and fourth carbon atoms. In the (+)*S*(+) conformation the current loops clockwise, in the (–)*S*(–) conformation it loops counter-clockwise. The current densities of the two conformations look like they are somewhat related by mirror-image symmetry, but it is the chirality of the electronic structure that is mirrored; the (+)*S*(+) and (–)*S*(–) conformations are not mirror images of each other. These circular currents are reminiscent of the ring currents found in cyclic π-conjugated molecules such as benzene, and we shall see there are more similarities with the currents in cyclic molecules.^81^–^83^

By inspection, the current density shown in Fig. 7 is clearly dominated by circular currents. When plotting the vector field on a dense grid it becomes apparent that there is a nodal plane, which can be seen from the side-view as the carbon atoms (grey balls) are visible through the arrows when their radius is very small. This is peculiar as the helical orbitals have helical nodal planes. The nodal planes in the current density of the (+)*S*(+) and (–)*S*(–) conformations are related by a 90° rotation around the *z*-axis, as can be seen by comparing the two rightmost columns of Fig. 7. This brings us back to the fact that the helical orbitals are a superposition of the rectilinear π-orbitals.^2^ While it is not obvious where the nodal plane in the current density forms, it is clear that the nodal planes have their origin from mutually orthogonal π-orbitals.

### Helical orbitals and circular currents

**Fig. 8:**
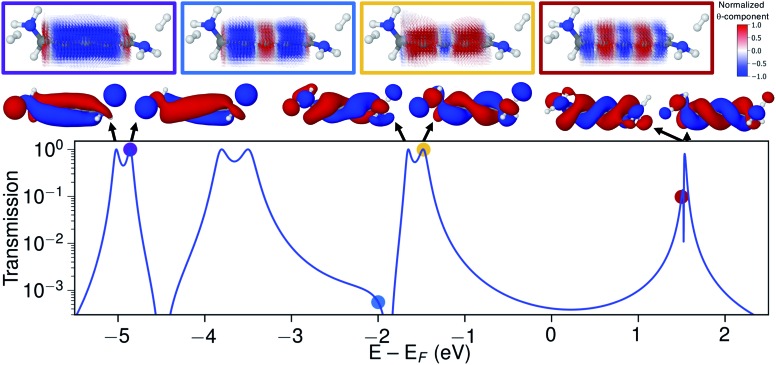
Transmission calculated with s-band electrodes, molecular orbitals of the junction, and current density calculated at select energies for the (–)*S*(–) conformation of S-1,5-diamino-[4]cumulene. The current density vector field is shown on a high-density grid and is colored by the *θ*-component. In the transmission plot, arrows indicate orbital energy (at the corresponding transmission resonances), colored dots indicate the energy at which the current density is calculated.

Let us now explore the link between electronic structure, electronic transmission, and current density. In Fig. 8 we show the transmission, molecular orbitals, and current density at select energies as calculated with s-band electrodes for the (–)*S*(–) conformation of 1,5-diamino-[4]cumulene. While previous figures showed the current density at the Fermi energy, the bias window is instead opened at specific energies in Fig. 8.

The current density calculated at the HOMO resonance (at –1.475 eV) looks similar to those at the Fermi energy (Fig. 8), however, with a circular current of the opposite direction at the central carbon atom. The number of times the circular currents change direction along the *z*-axis has an energy dependence. At higher energies, *e.g.* close to the LUMO resonance, the circular currents change direction more frequently as seen by the alternating colours in the current density. At lower energies, *e.g.* at –4.859 eV at the HOMO–4 resonance, there is no change of direction and the currents are almost perfectly circular along the full length of the wire.

Just below the HOMO–1 resonance energy, a sharp antiresonance appears in the transmission. Plotting the current density at an energy on the other side of the antiresonance (at –2.0 eV), we see a full reversal of the ring currents compared with that seen at the HOMO resonance. That is, the full current density field runs in the reverse circular direction. This ring-current reversal was also found in meta-linked benzene and is a clear signature of destructive quantum interference along with the antiresonance in the transmission.^84^

**Fig. 9:**
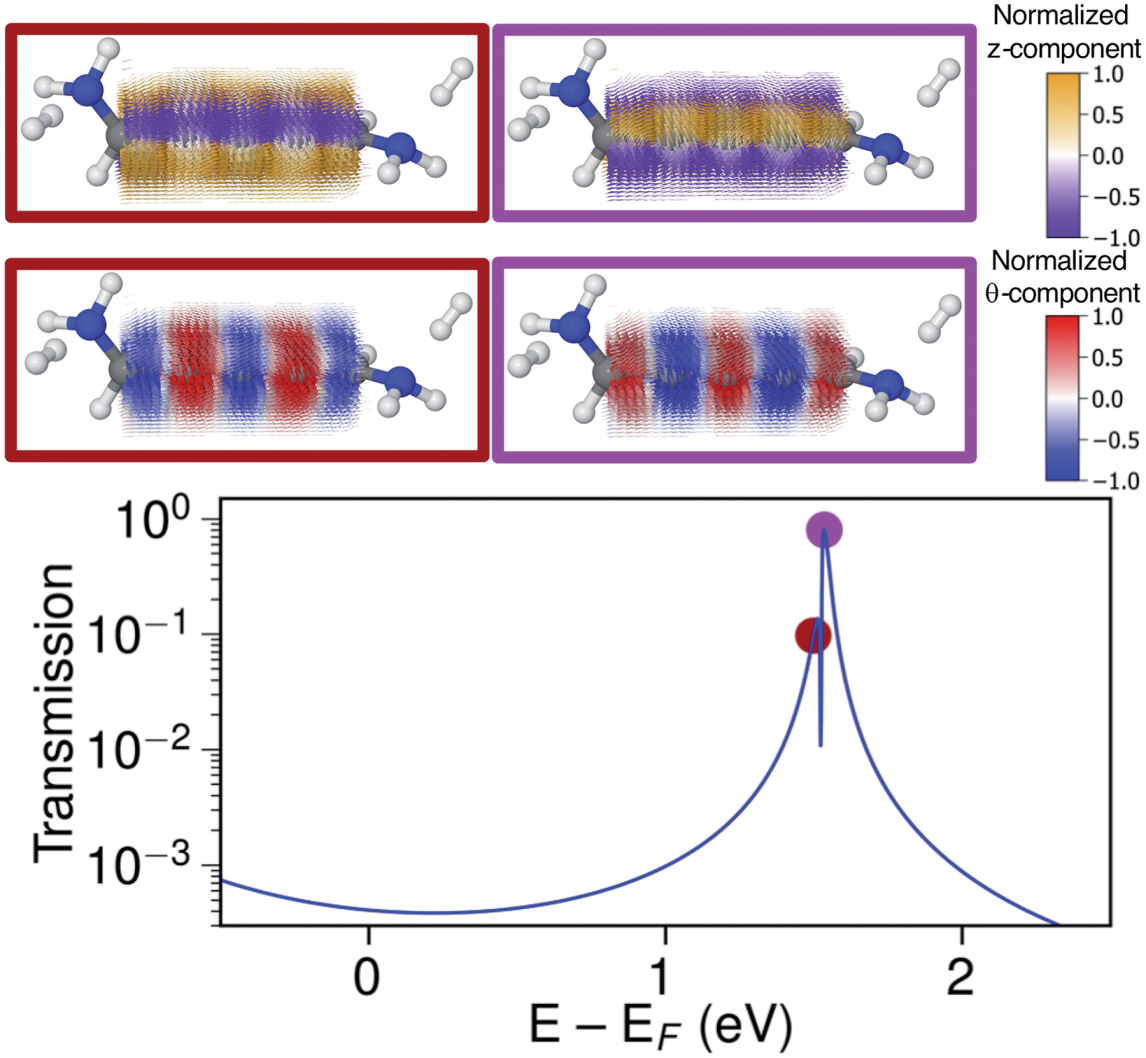
Transmission calculated with s-band electrodes and current density calculated around the LUMO resonance energy for the (–)*S*(–) conformation of 1,5-diamino-[4]cumulene. The current density vector field is shown on a high-density grid and is coloured by the *z*-component (first row) and by the *θ*-component (second row). In the transmission plot coloured dots indicate the energy at which the current density is calculated.

The ring-current reversal is also seen around the LUMO resonance where a narrow antiresonance appears as shown specifically in Fig. 9. The full reversal of circular currents is clear when colouring the vector field by the *θ*-component. However, the reversal effect is even more pronounced when colouring the vector field by the *z*-component. The current density has strong currents running against the direction of the total current; these currents also fully reverse at the antiresonance. The current densities through these linear wires are remarkably turbulent, a surprising manifestation of electrohelicity in the coherent transport properties of even [*n*]cumulenes.

### Circular currents in long wires

Though very long cumulenic wires are not realistic synthetic targets at the current time, we find it instructive to examine the behaviour of long wires. The nodal structure of the helical orbitals is fully preserved for longer wires, and the examination of the current density of [4]cumulene is expected to be general for the longer members of the series of even [*n*]cumulenes.

**Fig. 10:**
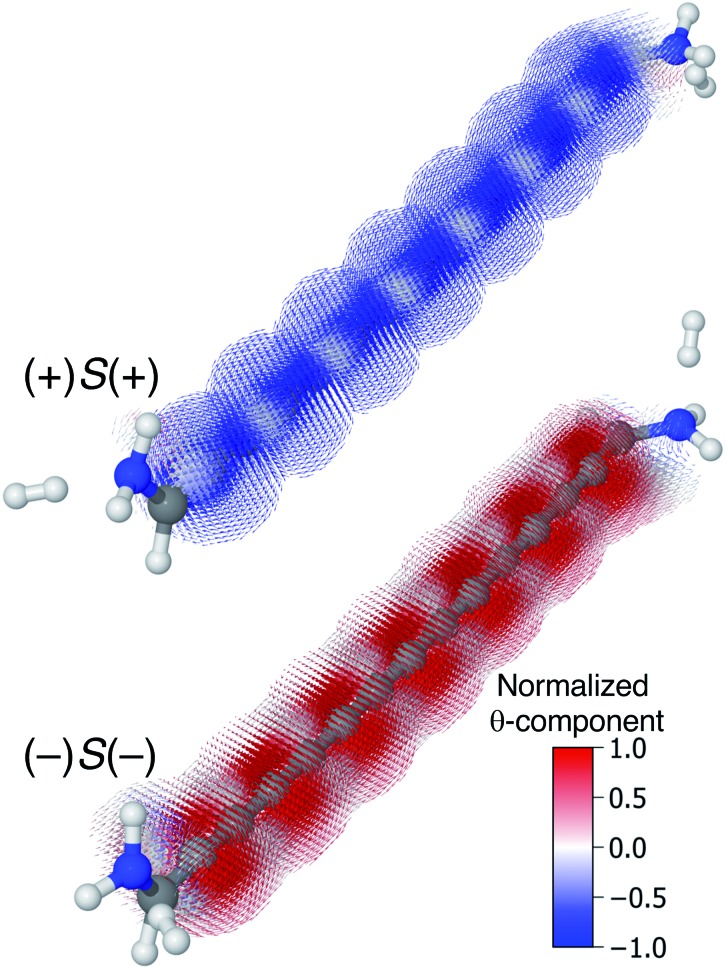
Current density calculated at the Fermi energy for the (+)*S*(+) and (–)*S*(–) conformations of 1,15-diamino-[14]cumulene; the 15-carbon cumulene with helical π-orbitals. The vector field is calculated on a high-density grid and coloured by the *θ*-component.

In Fig. 10 the current density at the Fermi energy is shown for the (+)*S*(+) and (–)*S*(–) conformations of 1,15-diamino-[14]cumulene. Just as the structure of the molecule is simply a long version of the shorter cumulenes, the current density patterns are identical to those of their shorter counterparts. There are strong circular currents of opposite directions in the (+)*S*(+) and (–)*S*(–) conformations. These circular currents are strongest around approximately every second carbon atom, and therefore seven current vortices are apparent in these [14]cumulenes. Considering it is the chirality of the amine groups at the termini that control this behaviour, it is remarkable that the circular current patterns are fully retained even in much longer wires.

## Conclusions

We have presented a general chemical strategy for splitting the otherwise degenerate helical frontier molecular orbitals of even [*n*]cumulenes. By using pyramidalized single-faced π-donors significant energetic splitting of the helical orbitals can be achieved, and it is the chiral centre at the lone-pair of such substituents that controls the helicity of the frontier orbitals. Consequently, we predict that helical orbitals will manifest themselves in experimental observables.

Here, we have demonstrated that the coherent electron transport properties of these cumulenic wires are directly controlled by the helicity and energetic splitting of the frontier orbitals. This allows us to explicitly predict the appearance of bimodal single-molecule conductance in single-molecule junction experiments. The current density through even [*n*]cumulenes is also dictated by their helical orbitals and strong circular currents are found along the wires. The direction of these currents is controlled by the chirality of the helical orbitals. We show that this is a manifestation of destructive quantum interference as the ring currents show full reversal around antiresonances in the transmission.

There are apparent perspectives in the finding of strong circular currents in linear carbon wires. Unidirectional ring currents will give rise to a magnetic field and even [*n*]cumulenes may therefore exhibit magnetoresistance.^85^–^87^ Such effects have recently been demonstrated in more complex molecular systems at room temperature,^88^–^91^ and would be remarkable to find in a truly linear atomic wire. Furthermore, if there is significant coupling to the electron spin [*n*]cumulenes could potentially function as spin filters.^92^–^95^ It is clear that the effect of helical electronic structure on the electron transport properties of linear wires must be explored further, and existing theories for describing transport through helical systems must be revisited in future research.^96^–^100^ Cumulene-based materials have been suggested and demonstrated in polymers;^101^–^103^ we envision the knowledge of helical electronic structure can form the basis for molecular devices and materials with novel electronic functionality.

## Conflicts of interest

There are no conflicts to declare.

## Supplementary Material

Supplementary information

## References

[cit1] Hendon C. H., Tiana D., Murray A. T., Carbery D. R., Walsh A. (2013). Chem. Sci..

[cit2] Garner M. H., Hoffmann R., Rettrup S., Solomon G. C. (2018). ACS Cent. Sci..

[cit3] Fischer H., Kollmar H. (1968). Theor. Chim. Acta.

[cit4] Herges R. (2015). J. Org. Chem..

[cit5] Shen L., Zeng M., Yang S.-W., Zhang C., Wang X., Feng Y. (2010). J. Am. Chem. Soc..

[cit6] Cretu O., Botello-Mendez A. R., Janowska I., Pham-Huu C., Charlier J.-C., Banhart F. (2013). Nano Lett..

[cit7] La Torre A., Botello-Mendez A., Baaziz W., Charlier J. C., Banhart F. (2015). Nat. Commun..

[cit8] Aoki Y., Orimoto Y., Imamura A. (2018). ACS Cent. Sci..

[cit9] Mustafa H. H., Baird M. S., Al Dulayymi J. a. R., Tverezovskiy V. V. (2013). Chem. Commun..

[cit10] Tiana D., Hendon C. H., Walsh A. (2014). Chem. Commun..

[cit11] Caricato M. (2015). J. Chem. Theory Comput..

[cit12] Sarbadhikary P., Shil S., Panda A., Misra A. (2016). J. Org. Chem..

[cit13] Gluyas J. B. G., Gückel S., Kaupp M., Low P. J. (2016). Chem. - Eur. J..

[cit14] Gückel S., Gluyas J. B. G., El-Tarhuni S., Sobolev A. N., Whiteley M. W., Halet J.-F., Lapinte C., Kaupp M., Low P. J. (2018). Organometallics.

[cit15] Imamura A., Aoki Y. (2013). Chem. Phys. Lett..

[cit16] Liu M., Artyukhov V. I., Lee H., Xu F., Yakobson B. I. (2013). ACS Nano.

[cit17] Guo Y.-D., Yan X.-H., Xiao Y. (2013). RSC Adv..

[cit18] Peeks M. D., Neuhaus P., Anderson H. L. (2016). Phys. Chem. Chem. Phys..

[cit19] Kuhn R., Fischer H., Fischer H. (1964). Chem. Ber..

[cit20] Ripoll J. L. (1976). J. Chem. Soc., Chem. Commun..

[cit21] Irngartinger H., Götzmann W. (1986). Angew. Chem., Int. Ed..

[cit22] Tokitoh N., Suzuki T., Ando W. (1989). Tetrahedron Lett..

[cit23] Bildstein B. (2000). Coord. Chem. Rev..

[cit24] Bildstein B., Skibar W., Schweiger M., Kopacka H., Wurst K. (2001). J. Organomet. Chem..

[cit25] Skibar W., Kopacka H., Wurst K., Salzmann C., Ongania K.-H., Fabrizi de Biani F., Zanello P., Bildstein B. (2004). Organometallics.

[cit26] Wendinger D., Tykwinski R. R. (2017). Acc. Chem. Res..

[cit27] Touchard D., Haquette P., Daridor A., Toupet L., Dixneuf P. H. (1994). J. Am. Chem. Soc..

[cit28] Dede M., Drexler M., Fischer H. (2007). Organometallics.

[cit29] Bildstein B., Kopacka H., Schweiger M., Ellmerer-Müller E., Ongania K.-H., Wurst K. (1996). Organometallics.

[cit30] Tzirakis M. D., Gisselbrecht J.-P., Boudon C., Trapp N., Diederich F. (2014). Tetrahedron.

[cit31] Zhang Y.-Q., Öner M. A., Lahoz I. R., Cirera B., Palma C.-A., Castro-Fernández S., Míguez-Lago S., Cid M. M., Barth J. V., Alonso-Gómez J. L., Klappenberger F. (2014). Chem. Commun..

[cit32] Ozcelik A., Pereira-Cameselle R., von Weber A., Paszkiewicz M., Carlotti M., Paintner T., Zhang L., Lin T., Zhang Y. Q., Barth J. V., van den Nobelen T., Chiechi R. C., Jakob M., Heiz U., Chiussi S., Kartouzian A., Klappenberger F., Alonso-Gómez J. L. (2018). Langmuir.

[cit33] Banhart F. (2015). Beilstein J. Nanotechnol..

[cit34] Franz M., Januszewski J. A., Wendinger D., Neiss C., Movsisyan L. D., Hampel F., Anderson H. L., Görling A., Tykwinski R. R. (2015). Angew. Chem., Int. Ed..

[cit35] Milan D. C., Krempe M., Ismael A. K., Movsisyan L. D., Franz M., Grace I., Brooke R. J., Schwarzacher W., Higgins S. J., Anderson H. L., Lambert C. J., Tykwinski R. R., Nichols R. J. (2017). Nanoscale.

[cit36] Larsen A. H., Mortensen J. J., Blomqvist J., Castelli I. E., Christensen R., Dułak M., Friis J., Groves M. N., Hammer B., Hargus C., Hermes E. D., Jennings P. C., Jensen P. B., Kermode J., Kitchin J. R., Kolsbjerg E. L., Kubal J., Kaasbjerg K., Lysgaard S., Maronsson J. B., Maxson T., Olsen T., Pastewka L., Peterson A., Rostgaard C., Schiøtz J., Schütt O., Strange M., Thygesen K. S., Vegge T., Vilhelmsen L., Walter M., Zeng Z., Jacobsen K. W. (2017). J. Phys.: Condens. Matter.

[cit37] Mortensen J. J., Hansen L. B., Jacobsen K. W. (2005). Phys. Rev. B: Condens. Matter Mater. Phys..

[cit38] Larsen A. H., Vanin M., Mortensen J. J., Thygesen K. S., Jacobsen K. W. (2009). Phys. Rev. B: Condens. Matter Mater. Phys..

[cit39] Perdew J. P., Burke K., Ernzerhof M. (1996). Phys. Rev. Lett..

[cit40] Jmol: an open-source Java viewer for chemical structures in 3D, http://www.jmol.org/.

[cit41] Chen J., Thygesen K. S., Jacobsen K. W. (2012). Phys. Rev. B: Condens. Matter Mater. Phys..

[cit42] Jensen A., Garner M. H. and Solomon G. C., When Current Does Not Follow Bonds: Current Density in Saturated Molecules, ChemRxiv. Preprint, 2019, 10.26434/chemrxiv.7851587.

[cit43] Garner M. H., Koerstz M., Jensen J. H., Solomon G. C. (2018). J. Phys. Chem. Lett..

[cit44] Datta S., Electronic Transport in Mesoscopic Systems, Cambridge University Press, 1995.

[cit45] Xue Y., Ratner M. A. (2004). Phys. Rev. B: Condens. Matter Mater. Phys..

[cit46] Tada T., Yoshizawa K. (2002). ChemPhysChem.

[cit47] Yoshizawa K., Tada T., Staykov A. (2008). J. Am. Chem. Soc..

[cit48] Härtle R., Butzin M., Rubio-Pons O., Thoss M. (2011). Phys. Rev. Lett..

[cit49] Ballmann S., Härtle R., Coto P. B., Elbing M., Mayor M., Bryce M. R., Thoss M., Weber H. B. (2012). Phys. Rev. Lett..

[cit50] Sowa J. K., Mol J. A., Briggs G. A. D., Gauger E. M. (2018). J. Phys. Chem. Lett..

[cit51] Tommasini M., Milani A., Fazzi D., Lucotti A., Castiglioni C., Januszewski J. A., Wendinger D., Tykwinski R. R. (2014). J. Phys. Chem. C.

[cit52] Jochims J. C., Karich G. (1974). Tetrahedron Lett..

[cit53] Jochims J. C., Karich G. (1976). Tetrahedron Lett..

[cit54] Nader F. W., Brecht A. (1986). Angew. Chem., Int. Ed..

[cit55] Suzuki N., Ohara N., Nishimura K., Sakaguchi Y., Nanbu S., Fukui S., Nagao H., Masuyama Y. (2011). Organometallics.

[cit56] Januszewski J. A., Wendinger D., Methfessel C. D., Hampel F., Tykwinski R. R. (2013). Angew. Chem., Int. Ed..

[cit57] Rauk A., Drake A. F., Mason S. F. (1979). J. Am. Chem. Soc..

[cit58] Elsevier C. J., Vermeer P., Gedanken A., Runge W. (1985). J. Am. Chem. Soc..

[cit59] Wiberg K. B., Wang Y.-g., Wilson S. M., Vaccaro P. H., Jorgensen W. L., Crawford T. D., Abrams M. L., Cheeseman J. R., Luderer M. (2008). J. Phys. Chem. A.

[cit60] Itatani J., Levesque J., Zeidler D., Niikura H., Pépin H., Kieffer J. C., Corkum P. B., Villeneuve D. M. (2004). Nature.

[cit61] Repp J., Meyer G., Stojković S. M., Gourdon A., Joachim C. (2005). Phys. Rev. Lett..

[cit62] Lüftner D., Ules T., Reinisch E. M., Koller G., Soubatch S., Tautz F. S., Ramsey M. G., Puschnig P. (2014). Proc. Natl. Acad. Sci. U. S. A..

[cit63] Wießner M., Hauschild D., Sauer C., Feyer V., Schöll A., Reinert F. (2014). Nat. Commun..

[cit64] Weiß S., Lüftner D., Ules T., Reinisch E. M., Kaser H., Gottwald A., Richter M., Soubatch S., Koller G., Ramsey M. G., Tautz F. S., Puschnig P. (2015). Nat. Commun..

[cit65] Cocker T. L., Peller D., Yu P., Repp J., Huber R. (2016). Nature.

[cit66] Pham B. Q., Gordon M. S. (2017). J. Phys. Chem. A.

[cit67] Su T. A., Neupane M., Steigerwald M. L., Venkataraman L., Nuckolls C. (2016). Nat. Rev. Mater..

[cit68] Prasongkit J., Grigoriev A., Wendin G., Ahuja R. (2010). Phys. Rev. B: Condens. Matter Mater. Phys..

[cit69] Garner M. H., Bro-Jørgensen W., Pedersen P. D., Solomon G. C. (2018). J. Phys. Chem. C.

[cit70] Stuyver T., Perrin M., Geerlings P., De Proft F., Alonso M. (2018). J. Am. Chem. Soc..

[cit71] Stuyver T., Fias S., Geerlings P., De Proft F., Alonso M. (2018). J. Phys. Chem. C.

[cit72] Venkataraman L., Park Y. S., Whalley A. C., Nuckolls C., Hybertsen M. S., Steigerwald M. L. (2007). Nano Lett..

[cit73] Andrews D. Q., Solomon G. C., Van Duyne R. P., Ratner M. A. (2008). J. Am. Chem. Soc..

[cit74] Garner M. H., Solomon G. C., Strange M. (2016). J. Phys. Chem. C.

[cit75] Liu X., Sangtarash S., Reber D., Zhang D., Sadeghi H., Shi J., Xiao Z.-Y., Hong W., Lambert C. J., Liu S.-X. (2016). Angew. Chem., Int. Ed..

[cit76] Tsuji Y., Staykov A., Yoshizawa K. (2011). J. Am. Chem. Soc..

[cit77] Tsuji Y., Stuyver T., Gunasekaran S., Venkataraman L. (2017). J. Phys. Chem. C.

[cit78] Walz M., Wilhelm J., Evers F. (2014). Phys. Rev. Lett..

[cit79] Wilhelm J., Walz M., Evers F. (2014). Phys. Rev. B: Condens. Matter Mater. Phys..

[cit80] Cabra G., Jensen A., Galperin M. (2018). J. Chem. Phys..

[cit81] Rai D., Hod O., Nitzan A. (2010). J. Phys. Chem. C.

[cit82] Nozaki D., Schmidt W. G. (2017). J. Comput. Chem..

[cit83] Fias S., Stuyver T. (2017). J. Chem. Phys..

[cit84] Solomon G. C., Herrmann C., Hansen T., Mujica V., Ratner M. A. (2010). Nat. Chem..

[cit85] Hod O., Rabani E., Baer R. (2006). Acc. Chem. Res..

[cit86] Rai D., Hod O., Nitzan A. (2011). J. Phys. Chem. Lett..

[cit87] Rai D., Hod O., Nitzan A. (2012). Phys. Rev. B: Condens. Matter Mater. Phys..

[cit88] Hayakawa R., Karimi M. A., Wolf J., Huhn T., Zöllner M. S., Herrmann C., Scheer E. (2016). Nano Lett..

[cit89] Xie Z., Shi S., Liu F., Smith D. L., Ruden P. P., Frisbie C. D. (2016). ACS Nano.

[cit90] Shi S., Xie Z., Liu F., Smith D. L., Frisbie C. D., Ruden P. P. (2017). Phys. Rev. B.

[cit91] Aragonès A. C., Aravena D., Valverde-Muñoz F. J., Real J. A., Sanz F., Díez-Pérez I., Ruiz E. (2017). J. Am. Chem. Soc..

[cit92] Naaman R., Waldeck D. H. (2012). J. Phys. Chem. Lett..

[cit93] Naaman R., Waldeck D. H. (2015). Annu. Rev. Phys. Chem..

[cit94] Aragonès A. C., Medina E., Ferrer-Huerta M., Gimeno N., Teixidó M., Palma J. L., Tao N., Ugalde J. M., Giralt E., Díez-Pérez I., Mujica V. (2016). Small.

[cit95] Sarbadhikary P., Shil S., Misra A. (2018). Phys. Chem. Chem. Phys..

[cit96] Skourtis S. S., Beratan D. N., Naaman R., Nitzan A., Waldeck D. H. (2008). Phys. Rev. Lett..

[cit97] Yeganeh S., Ratner M. A., Medina E., Mujica V. (2009). J. Chem. Phys..

[cit98] Gutierrez R., Díaz E., Naaman R., Cuniberti G. (2012). Phys. Rev. B: Condens. Matter Mater. Phys..

[cit99] Maslyuk V. V., Gutierrez R., Dianat A., Mujica V., Cuniberti G. (2018). J. Phys. Chem. Lett..

[cit100] Qi D., Kenaan A., Cui D., Song J. (2018). Nano Energy.

[cit101] Diederich F. (1994). Nature.

[cit102] Rivera-Fuentes P., Alonso-Gómez J. L., Petrovic A. G., Santoro F., Harada N., Berova N., Diederich F. (2010). Angew. Chem., Int. Ed..

[cit103] Rivera-Fuentes P., Diederich F. (2012). Angew. Chem., Int. Ed..

