# Supplementary material for: Helical orbitals and circular currents in linear carbon wires
Source: Chem Sci. 2019 Mar 19;10(17):4598–608. doi: 10.1039/c8sc05464a (PMC6496982; doi:10.1039/c8sc05464a)
Supplement: Supplementary file 1 [file SC-010-C8SC05464A-s001.pdf]

Electronic Supplementary Material (ESI) for Chemical Science.
This journal is © The Royal Society of Chemistry 2019

Supplementary Information for: Helical Orbitals and Circular Currents in Linear Carbon Wires

*Marc H. Garner, Anders Jensen, Louise Hyllested, Gemma C. Solomon**

Department of Chemistry and Nano-Science Center, University of Copenhagen, Universitetsparken 5, DK-2100, Copenhagen Ø, Denmark.

Table of contents

I. Orbital Splitting and Zeroth-order Green's Function 2

II. Substituent Effects in 1,5-disubstitued [4]cumulenes 4

III. Substituent Effect of Pyramidalized Single-faced π-donors 5

IV. Aryl-substituted Cumulene 9

VI. Barriers between Conformations 12

V. [5]cumulene Transmission 13

VII. Coordinate Transformation and Current Density Convergence 14

VIII. Wide-band Transmission Plots 16

References 17

I. Orbital splitting and zeroth order Green's Function

The helical molecular orbitals of even [n]cumulenes can be described with a simple Hückel model, which we have done in previous work.[1]

Within the Hückel model, the Landauer transmission can be predicted explicitly by analysing the zeroth-order Green's function, G^0, which when squared is proportional to the transmission.[2, 3]

$$T(E) \propto G^0(E)^2 \qquad \text{(S1)}$$

$$G^0(E) = \sum_i \frac{c_{i,L} \cdot c_{i,R}}{\epsilon_i - E} \qquad \text{(S2)}$$

G^0 is a sum over all molecular orbitals, and c_L and c_R are the orbital coefficients of the ith molecular orbital at the injection points of the left and right electrodes.

If we look at just one pair of degenerate orbitals, e.g. the HOMO and HOMO–1 of an even [n]cumulene, then we can write out the summation.

$$G^0_{HOMO/HOMO-1}(E) = \frac{c_{HOMO,L} \cdot c_{HOMO,R}}{\epsilon_{HOMO} - E} + \frac{c_{HOMO-1,L} \cdot c_{HOMO-1,R}}{\epsilon_{HOMO-1} - E} \qquad \text{(S3)}$$

Now let us assume that the magnitude of the c coefficient is the same in all cases, as is explicitly the case in D_{2d} symmetry cumulenes. Then it is only a matter of the sign of the product of the c coefficients. For even [n]cumulenes the sign of the two products will be different (see Figure 1 and Figure 4 in the manuscript). Using these assumptions, we achieve a proportionality for the Green's function of the HOMO and HOMO–1, which we can simplify.

$$G^0_{HOMO/HOMO-1}(E) \propto \frac{1}{\epsilon_{HOMO} - E} - \frac{1}{\epsilon_{HOMO-1} - E} \qquad \text{(S4)}$$

$$G^0_{HOMO/HOMO-1}(E) \propto \frac{\epsilon_{HOMO-1} - E}{(\epsilon_{HOMO} - E)(\epsilon_{HOMO-1} - E)} - \frac{\epsilon_{HOMO} - E}{(\epsilon_{HOMO-1} - E)(\epsilon_{HOMO} - E)}$$

$$G^0_{HOMO/HOMO-1}(E) \propto \frac{\epsilon_{HOMO-1} - \epsilon_{HOMO}}{(\epsilon_{HOMO} - E)(\epsilon_{HOMO-1} - E)}$$

It follows from this that the contribution to the zeroth-order Green's function from the HOMO and HOMO–1 is proportional to the energy-splitting between these otherwise degenerate molecular orbitals.

$$G^0_{HOMO/HOMO-1}(E) \propto \epsilon_{HOMO-1} - \epsilon_{HOMO} \qquad \text{(S5)}$$

This proportionality is not energy dependent, and the contribution to the total transmission is therefore expected to be proportional to the square of the splitting. There is a caveat beyond the approximations mentioned above. The zeroth-order Green's function is a sum over all molecular orbitals, and transmission is therefore a coherent property of the full electronic structure. Definitive conclusions cannot generally be made by studying select molecular orbitals, though there are systems where this is justifiable if they follow the Coulson-Rushbrooke pairing theorem.[4, 5]

As a simple prediction though, it is clear that the splitting of the otherwise near-degenerate helical molecular orbital pairs is essential for the Landauer transmission. We verify this prediction in the manuscript using density functional theory.

II. Substituent effects in 1,5-disubstitued [4]cumulenes

In this section we explore substituent effects in 1,5-disubstituted [4]cumulenes. All calculations in this section were carried out using Gaussian09 using density functional theory (DFT) at the M06-2X/6-311G(d,p) level with the *verytight* optimization setting and *ultrafine* integral grid.[6,7] In Table S1 we list the helicity of the frontier orbitals for a range of substituents sorted by their Hammett constants,[8] while we plot their frontier orbitals splittings in Table S2. There is no appreciable tuning of the frontier orbitals with the listed substituents. The orbitals are all split in the same order; the electron withdrawing and donating substituents do not split the orbital pairs oppositely. Thus, the helicity of the HOMO is the same for all *S*-enantiomers. With exception of the most electron withdrawing substituents the effect is very weak. The HOMO and HOMO–1 of *S*-1,5-dichloro-[4]cumulene are split by 3 meV with M06-2X functional, which compares well with calculations with the PBE functional at 6 meV.

Table S1. Helicity (chirality) of frontier orbitals of *S*-1,5-diX-[4]cumulenes, sorted by their Hammett constants (σ_p) from electron withdrawing to electron donating.

X<br>σ_p	*-CN*<br>0.66	*-COOH*<br>0.45	*-Br*<br>0.23	*-F*<br>0.06	*CH_3*<br>–0.17	*-OCH_3*<br>–0.27	*-OH*<br>–0.37
LUMO+1	*P*	*P*	*P*	*P*	*P*	*P*	*P*
LUMO	*M*	*M*	*M*	*M*	*M*	*M*	*M*
HOMO	*P*	*P*	*P*	*P*	*P*	*P*	*P*
HOMO–1	*M*	*M*	*M*	*M*	*M*	*M*	*M*

Table S2. Frontier orbital splitting of disubstituted [4]cumulenes. Given as absolute energy difference in meV.

X<br>σ_p	*-CN*<br>0.66	*-COOH*<br>0.45	*-Br*<br>0.23	*-F*<br>0.06	*CH_3*<br>–0.17	*-OCH_3*<br>–0.27	*-OH*<br>–0.37
LUMO+1<br>LUMO	*7*	*27*	*2*	*4*	*1*	*2*	*5*
HOMO<br>HOMO–1	*7*	*19*	*2*	*3*	*0*	*3*	*2*

III. Substituent effect of Pyramidalized Single-faced π-donors

Pyramidalized single-faced π-donor substituents like the amine group provide a much stronger splitting of the helical orbitals and systematically tunes their helicity, as listed in Table S3 and S4 and described in the manuscript. In the case of (+)*S*(–), the symmetry is reduced to C_1 and, as shown in Figure S1, it is not clear by inspection if the orbitals are helical; it should be noted they are not split significantly and therefore should behave much like rectilinear π-orbitals.

Table S3. Helicity (chirality) of frontier orbitals of 1,5-substituted [4]cumulenes (GPAW: PBE/dzp)

	**NH_2** (+)*S*(+)	**NH_2** (–)*S*(–)	**NH_2** (+)*S*(–)	**Cl**
LUMO+1	*P*	*M*	n/a	*P*
LUMO	*M*	*P*	n/a	*M*
HOMO	*M*	*P*	n/a	*P*
HOMO–1	*P*	*M*	n/a	*M*

Table S4. Frontier orbital splitting of 1,5-disubstituted [4]cumulenes. Given as absolute energy difference in meV. (GPAW: PBE/dzp)

	**NH_2** (+)*S*(+)	**NH_2** (–)*S*(–)	**NH_2** (+)*S*(–)	**Cl**
LUMO+1 LUMO	37	49	2	10
HOMO HOMO–1	78	76	5	6

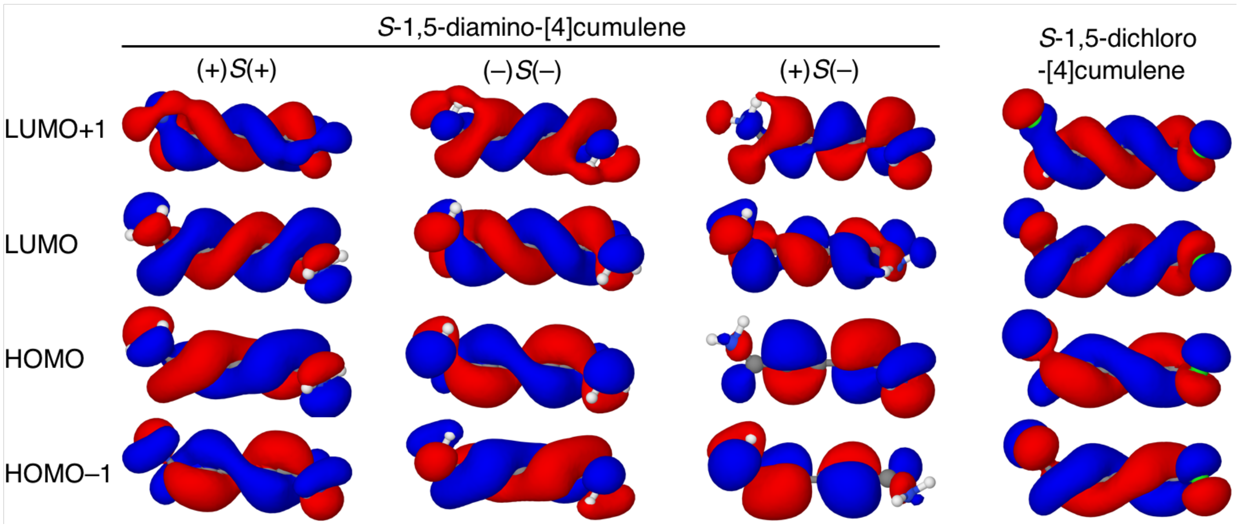

Figure S1. Frontier orbitals of *S*-1,5-diamino-[4]cumulene and *S*-1,5-dichloro-[4]cumulene as calculated with the PBE functional using GPAW.

DFT cannot be used to systematically describe virtual orbitals, and the order (the helicity) of the unoccupied orbitals is therefore not well-defined. In Figure S2 the frontier orbitals of *S*-1,5-diamino-[4]cumulene are calculated using Hartree-Fock/6-311G(d,p) as implemented in Gaussian09. With HF the conclusions from the manuscript remain. The helicity is systematically tuned by the conformation of the amine groups. Like with DFT-PBE, the HOMO of (+)*S*(+) is an *M* helix, and the HOMO of (–)*S*(–) is a *P* helix. However, the helicity of the LUMO is reversed compared to DFT-PBE; Within HF theory the LUMO of (+)*S*(+) is a *P* helix, and the HOMO of (–)*S*(–) is an *M* helix.

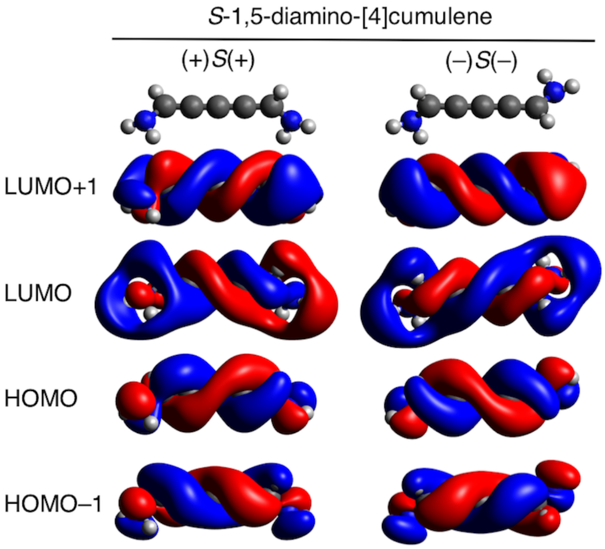

Figure S2. Frontier orbitals of *S*-1,5-diamino-[4]cumulene as calculated with Hartree-Fock.

Other pyramidalized single-faced π-donors include the $N(CH_3)_2$ and PH_2 substituents. In the remaining part of this section all calculations were performed using Gaussian09 with DFT at the M06-2X/6-311G(d,p) level with the *verytight* optimization setting and *ultrafine* integral grid.[6, 7]

The first thing to notice in Table S5 is that for the amine substituent the helicities of the unoccupied orbitals agrees with the HF calculations. This may both be a question of how well DFT methods describe the unoccupied orbitals, and the fact that the unoccupied orbitals are only split by a small amount. The HOMO and HOMO–1 are split the same *way* with the dimethylamino and phosphano substituents as described in the manuscript for the diamine substituent. The energy splittings of the frontier orbitals listed in Table S6 are all relatively large, and in the case of phospine significantly larger than for the amine substituent. These results suggest that the effect of having a chiral lone-pair is systematic and can be used for rational chemical design of molecules for utilizing the electrohelicity effect.

Table S5. Helicity (chirality) of frontier orbitals of the conformations of 1,5-diamino-[4]cumulene, 1,5-bis(dimethylamino)-[4]cumulene, and 1,5-diphosphino-[4]cumulene. (Gausian09: M06-2X/6-311G(d,p))

	$-NH_2$			$-NMe_2$			$-PH_2$		
	(+)*S*(+)	(–)*S*(–)	(+)*S*(–)	(+)*S*(+)	(–)*S*(–)	(+)*S*(–)	(+)*S*(+)	(–)*S*(–)	(+)*S*(–)
LUMO+1	*M*	*P*	*P*	*P*	*M*	*P*	*P*	*M*	*P*
LUMO	*P*	*M*	*M*	*M*	*P*	*M*	*M*	*P*	*M*
HOMO	*M*	*P*	n/a	*M*	*P*	*P*	*M*	*P*	n/a
HOMO–1	*P*	*M*	n/a	*P*	*M*	*M*	*P*	*M*	n/a

Table S6. Frontier orbital splitting of 1,5-diamino-[4]cumulene, 1,5-bis(dimethylamino)-[4]cumulene, and 1,5-diphosphino-[4]cumulene. Given as absolute energy difference in meV. (Gausian09: M06-2X/6-311G(d,p))

	$-NH_2$			$-NMe_2$			$-PH_2$		
	(+)*S*(+)	(–)*S*(–)	(+)*S*(–)	(+)*S*(+)	(–)*S*(–)	(+)*S*(–)	(+)*S*(+)	(–)*S*(–)	(+)*S*(–)
LUMO+1<br>LUMO	14	12	6	86	81	4	181	177	5
HOMO<br>HOMO-1	97	99	3	148	151	2	461	430	6

IV. Aryl-substituted Cumulene

We have found that other substituents with a *tilted* orbital system have a similar systematic tuning effect on the helical frontier orbitals. This may not be surprising, as such tilting makes the substituent a chiral centre, and thus the helical frontier orbitals on the cumulene can be tuned. Phenyl groups are one such group of substituents because the ortho hydrogens are sterically hindered and thus the phenyl tilts approximately ±30° out of the cumulene plane. As the phenyl tilt towards a positive or negative dihedral angle (arbitrarily defined from a phenyl carbon), three similar conformations to the amine case arise. We note there can be other conformations as well, and for a specific aryl substituent of interest a conformational search must be performed.

In Table S7 and Figure S3 we report the frontier orbitals of *S*-1,5-diphenyl-1,5-di-*t*-butyl-[4]cumulene as calculated with Gaussian09 using DFT at the M06-2X/6-311G(d,p) level with the *verytight* optimization setting and *ultrafine* integral grid.[6,7] The HOMO and HOMO–1 are significantly split, and as the case was with pyramidalized single-faced π-donors the conformation controls the helicity of the orbitals.

Table S7. Energy splitting of the HOMO and HOMO–1 of *S*-1,5-diphenyl-1,5-di-*t*-butyl-[4]cumulene. Given as absolute energy difference in meV.

	(+)*S*(+)	(–)*S*(–)	(+)*S*(–)
HOMO<br>HOMO-1	290	288	9

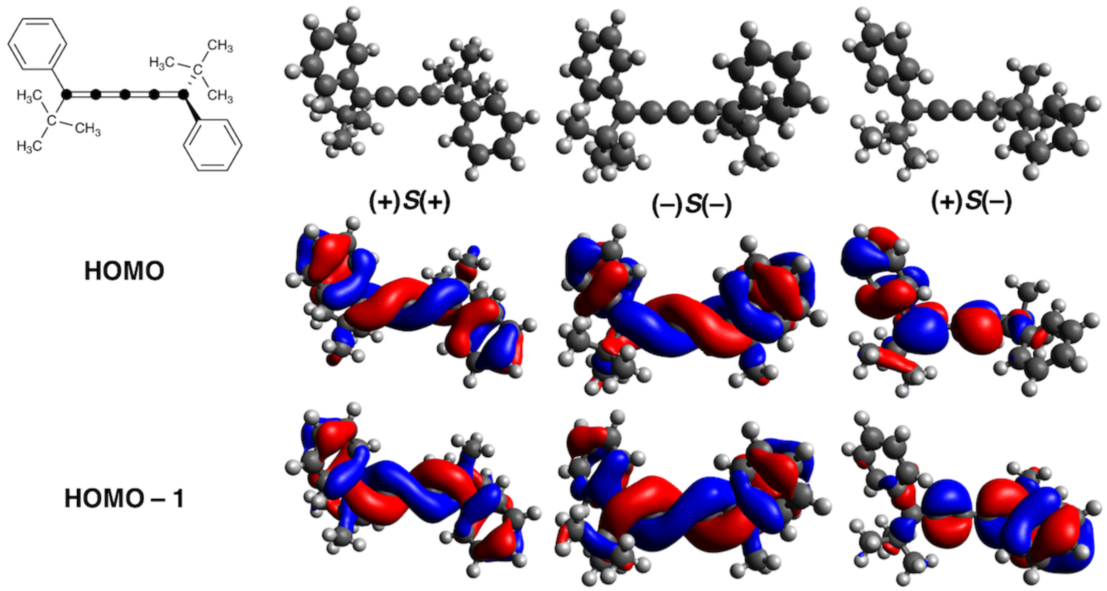

Figure S3. HOMO and HOMO–1 of (+)*S*(+) , (–)*S*(–) and (+)*S*(–) conformations *S*-1,5-diphenyl-1,5-di-*t*-butyl-[4]cumulene The HOMO of (+)*S*(+) is an *M*-helix and the HOMO–1 is a *P*-helix; the order is reversed for the (–)*S*(–) conformation.

Aryl substituents may hold promise for further functionalization due to their inherent stability. Furthermore, this means that the frontier orbital splitting is not controlled by the anchoring groups. Consequently, any anchoring group can in principle be used. In Figure S4 the Landauer transmission is calculated using DFT-PBE (GPAW) for the three conformations functionalized with thiols at the para positions. While the transmission is high for the (+)*S*(+) and (–)*S*(–) conformations, it is suppressed for the (+)*S*(–) conformation by approximately an order of magnitude.

Aryl substituted cumulenes may hold great promise as the conformations may be tuned further with substituents at the meta and ortho positions of the phenyl group. [9-14] As we discuss in the next section, such functionalization may furthermore be used to achieve larger energy barriers between conformations.

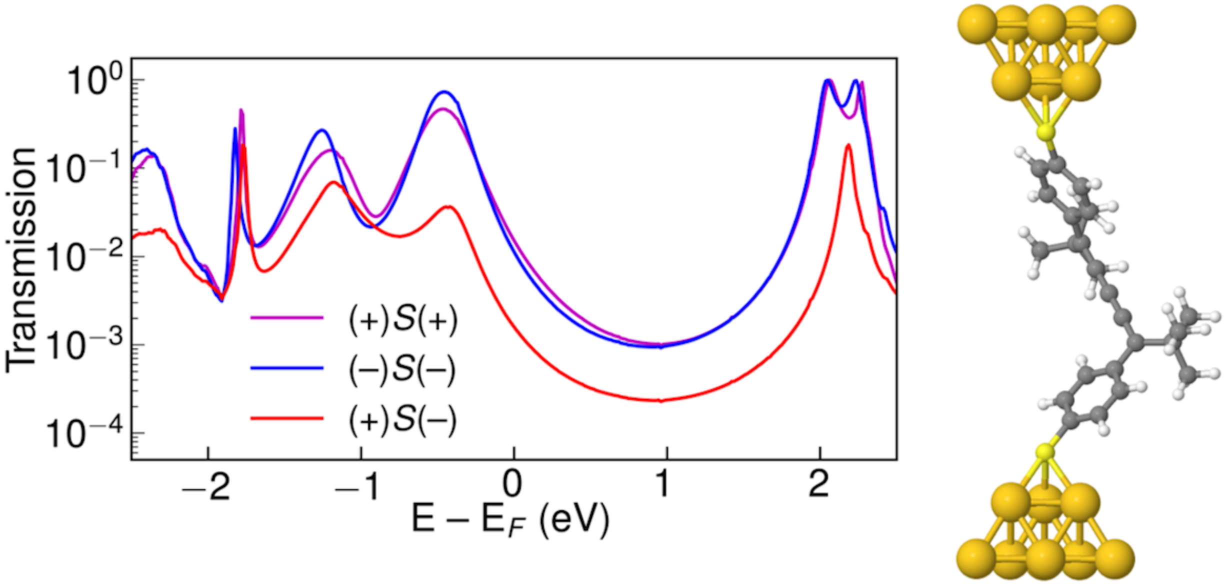

Figure S4. Left: Transmission of thiolated *S*-1,5-diphenyl-1,5-di-*t*-butyl-[4]cumulene plotted semilogarithmically against energy. Right: Optimized junction structure for (+)S(+) conformation, only tip Au atoms are shown for clarity.

V. [5]cumulene transmission

In this section, we show the Landauer transmission of the four conformations of 1,6-diamino-[5]cumulene in Au-junctions as calculated with GPAW. While there are clear differences in the transmission of the four, the difference of the transmission at the Fermi energy is small, approximately a factor of two between the highest and lowest transmission conformation (Cis ds and Trans ds).

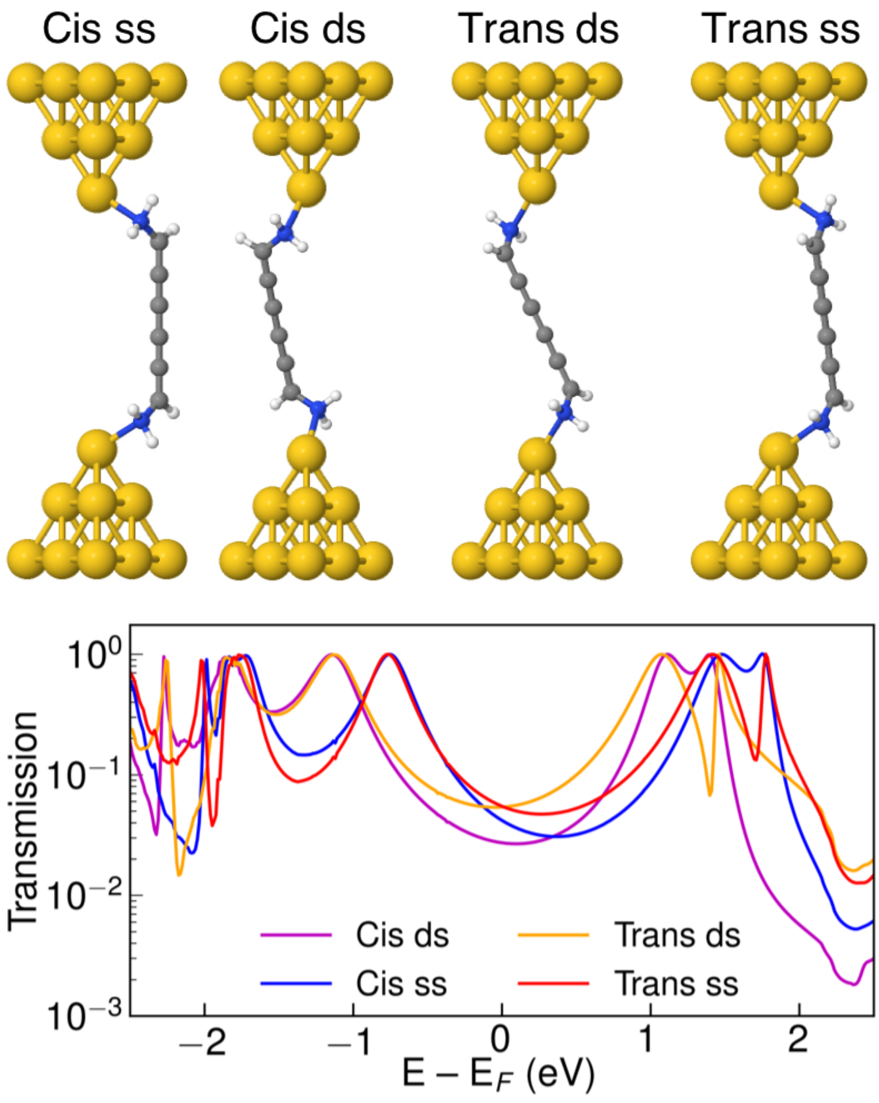

Figure S5. Transmission and optimized junction structures of 1,6-diamino-[5]cumulene. The cis and trans isomers can have lone-pairs pointing to the same side (ss) or to different sides (ds) of the plane of the cumulene.

VI. Barriers between Conformations

Let us examine the energy barrier through the transitions states (TS) connecting the conformations of *S*-1,5-diamino-[4]cumulene. There are two reaction paths for interconversion between the conformations. One where the hydrogens form a plane with the nitrogen and carbons atoms. Intuitively, we can describe this TS as one where the nitrogen lone-pair becomes a non-hybridized p-orbital in the TS. The alternative TS is one where the lone-pair moves as the hydrogens rotate around the C=C-N-H dihedral angle through a TS where the lone-pair lies in the plane of the molecule.

Conversion from (–)*S*(–) to (+)*S*(–) calculated using M06-2X/6-311G(d,p). The interconversion path through TS1 (Figure S6, planar) is almost barrierless at 7 meV. However, this interconversion path will not be available when the molecule is in a single-molecule junction (or a similar device) because a large metal atom will be bound to the nitrogen lone-pair. The interconversion path through TS2 (Figure S6, torsional) is however more notable at 316 meV. While still not a huge barrier, it is approaching a size where conformations may exist on a microsecond scale. It is therefore expected that the different conformations can be measured as separable signals in appropriate experimental setups. Larger substituents are likely to have larger interconversion barriers, and future work is to be directed at finding promising synthetic targets.

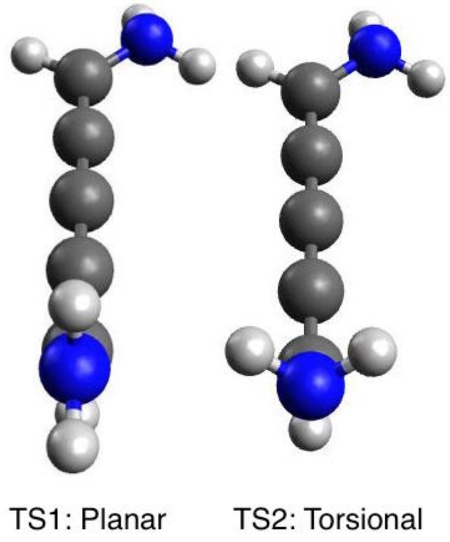

Figure S6. Planar TS and torsional TS between the lone-pair conformations of *S*-1,5-diamino-[4]cumulene.

VII. Coordinate Transformation and Current Density Convergence

A conversion from cartesian coordinates is trivial and essentially converts cartesian *x,y*-coordinates into polar *r,θ*-coordinates while the *z*-coordinate is unchanged.[15] The polar (cylindrical) vector components can be calculated as

$$\vec{v}_{r,\theta} = \left(v_x \cdot \cos(\theta) + v_y \cdot \sin(\theta)\right) \cdot \hat{e}_r + \left(v_y \cdot \cos(\theta) - v_x \cdot \sin(\theta)\right) \cdot \hat{e}_\theta , \quad \text{(S6)}$$

where $\hat{e}_r$ and $\hat{e}_\theta$ are the cylindrical unit vectors, and v_x and v_y are the cartesian vector components. The carbon axis is aligned with the *z*-axis placing it in the origin of the *r* and θ dimensions, as shown in Figure 2 in the manuscript. Our current density code produces the current density vector field colored by the z-component and by the θ-component, and is available for free at https://github.com/marchgarner/Current_Density.git

As described in recent work,[16] the current density may not preserve the total current throughout the molecule due to the finite local basis set that is used in the calculation. Such error can be estimated by integrating the current density **j(r)** over a plane **A** perpendicular to the transport direction *z*.

$$J = \int \mathbf{j}(\mathbf{r}) d\mathbf{A},\ d\mathbf{A} = dxdy \quad \text{(S7)}$$

The current through the plane *J* can then be compared with the total current, here denoted *I*, which can be calculated using the general Landauer formula

$$I = \frac{e\hbar}{2\pi} \int dE \left(f_L(E) - f_R(E)\right) \cdot T(E), \quad \text{(S8)}$$

where *T(E)* is the transmission function and $f_{L/R}$ are the fermi functions of the left and right electrodes. The two approaches are equivalent, so *J* should equal *I* at any chosen area. In Figure S7 and S8 the current is plotted as a function of the z-coordinate in the junctions of (–)S(–) and (+)S(+) 1,5-diamino-[4]cumulene. The dashed red line shows the total current as calculated with the Landauer formula, which is constant throughout the junction. Around the nitrogen atoms, where the injection happens the current spikes to around twice the total current, while at each of the five carbon atoms the current is slightly below the total current. At the edges of the *box* the current drops to zero.

It is clear that the current density fluctuates around the nitrogen atoms, which may be due to being close to the edge of the box. However, the current density is reasonably converged at the cumulenic carbon atoms. Therefore, we only plot the current density starting at the first carbon atom of the cumulene chain and until the last carbon atom of the chain.

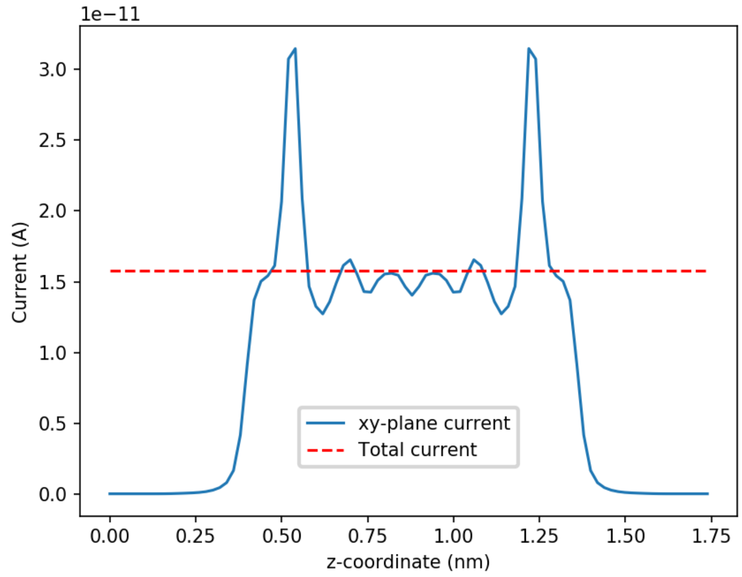

Figure S7. Total current and integrated current from the current density as function of *z*-coordinate (the transport direction) calculated for (–)S(–) junction 1,5-diamino-[4]cumulene. The nitrogen atoms are at 0.53 nm and 1.23 nm.

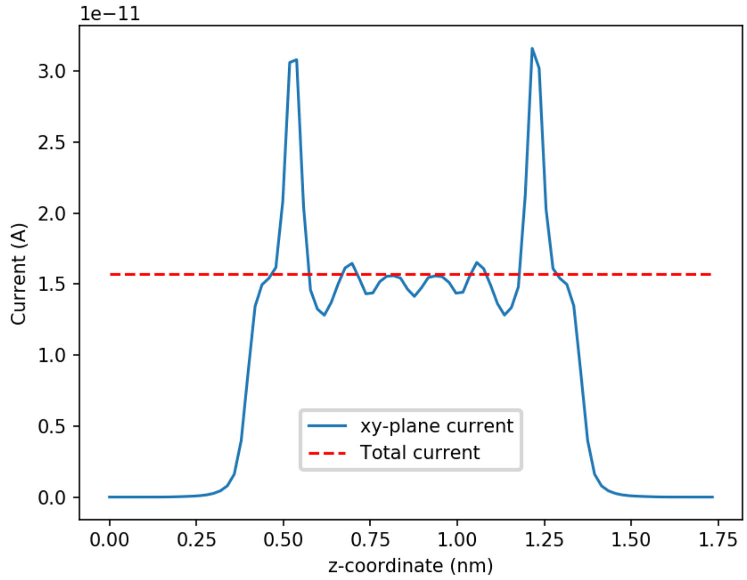

Figure S8. Total current and integrated current from the current density as function of z-coordinate (the transport direction) calculated for (+)S(+) junction 1,5-diamino-[4]cumulene. The nitrogen atoms are at 0.53 nm and 1.22 nm.

VIII. Wide-band Transmission Plots

In Figure S9, we show the current density and transmission calculated using wide-band electrodes for [5]cumulene. As one would expect, all four conformations show predominantly linear currents. The transmission of the four conformations is nearly identical around the Fermi energy.

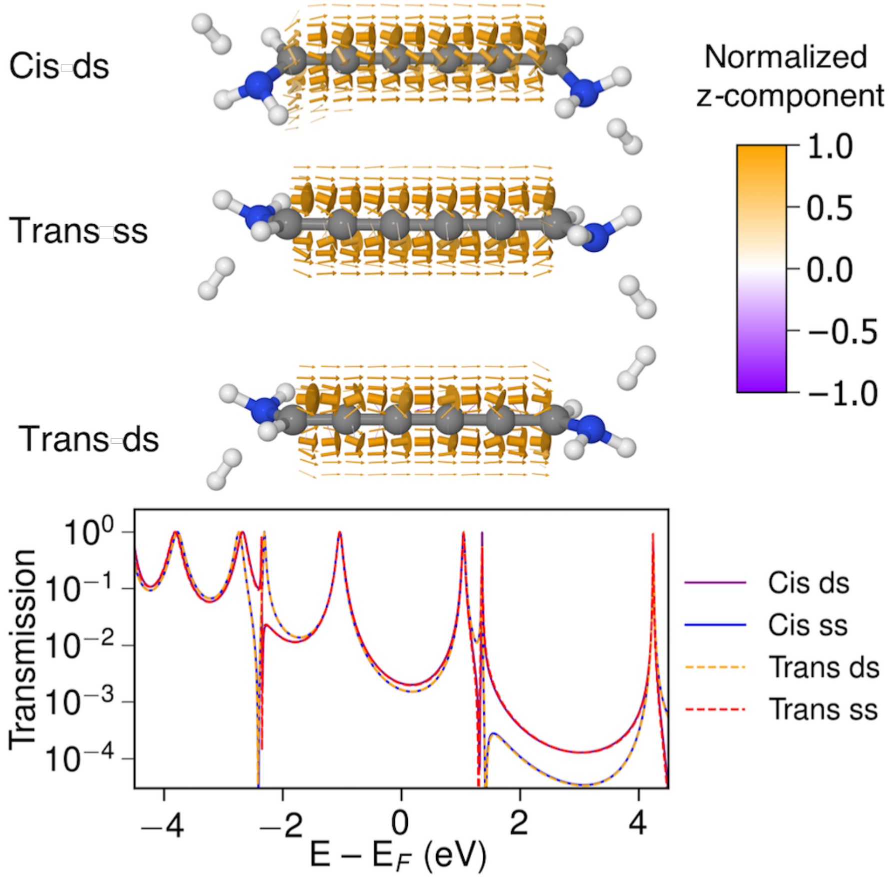

Figure S9. Current density and transmission of 1,6-diamino-[5]cumulene calculated using wide-band electrodes. The cis and trans isomers can have lone-pairs pointing to the same side (ss) or to different sides (ds) of the plane of the cumulene. The current density of Cis-ss is shown in Figure 6 in the manuscript

As the case is when using Au-electrodes, the transmission of [4]cumulene is significantly split. The (+)*S*(+) and (–)*S*(–) conformations have high transmission, while the (+)*S*(–) conformation has very low transmission. The suppression of the transmission is clearly due to destructive quantum interference, and an antiresonance is present at around –1 eV for in the (+)*S*(–) conformation.

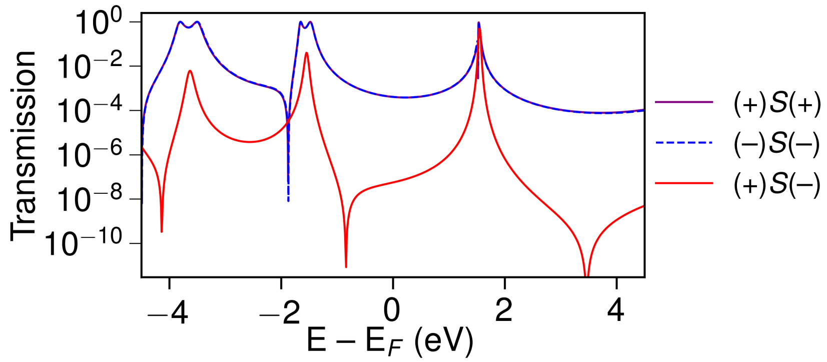

Figure S10. Transmission of 1,5-diamino-[4]cumulene calculated using wide-band electrodes.

REFERENCES

1. M. H. Garner, R. Hoffmann, S. Rettrup and G. C. Solomon, *ACS Cent. Sci.*, 2018, **4**, 688-700.
2. T. Tada and K. Yoshizawa, *ChemPhysChem*, 2002, **3**, 1035-1037.
3. K. Yoshizawa, T. Tada and A. Staykov, *J. Am. Chem. Soc.*, 2008, **130**, 9406-9413.
4. C. A. Coulson and G. S. Rushbrooke, *Math. Proc. Camb. Philos. Soc.*, 1940, **36**, 193-200.
5. X. Zhao, V. Geskin and R. Stadler, *J. Chem. Phys.*, 2016, **146**, 092308.
6. M. J. Frisch, G. W. Trucks, H. B. Schlegel, G. E. Scuseria, M. A. Robb, J. R. Cheeseman, G. Scalmani, V. Barone, B. Mennucci, G. A. Petersson, H. Nakatsuji, M. Caricato, X. Li, H. P. Hratchian, A. F. Izmaylov, J. Bloino, G. Zheng, J. L. Sonnenberg, M. Hada, M. Ehara, K. Toyota, R. Fukuda, J. Hasegawa, M. Ishida, T. Nakajima, Y. Honda, O. Kitao, H. Nakai, T. Vreven, J. J. A. Montgomery, J. E. Peralta, F. Ogliaro, M. Bearpark, J. J. Heyd, E. Brothers, K. N. Kudin, V. N. Staroverov, T. Keith, R. Kobayashi, J. Normand, K. Raghavachari, A. Rendell, C. Burant, S. S. Iyengar, J. Tomasi, M. Cossi, N. Rega, J. M. Millam, M. Klene, J. E. Knox, J. B. Cross, V. Bakken, C. Adamo, J. Jaramillo, R. Gomperts, R. E. Stratmann, O. Yazyev, A. J. Austin, R. Cammi, C. Pomelli, J. W. Ochterski, R. L. Martin, K. Morokuma, V. G. Zakrzewski, G. A. Voth, P. Salvador, J. J. Dannenberg, S. Dapprich, A. D. Daniels, O. Farkas, J. B. Foresman, J. V. Ortiz, J. Cioslowski and D. J. Fox, *Journal*, 2013.

7. Y. Zhao and D. G. Truhlar, *Theor. Chem. Acc.*, 2008, **120**, 215-241.
8. C. Hansch, A. Leo and R. W. Taft, *Chem. Rev.*, 1991, **91**, 165-195.
9. R. Kuhn, B. Schulz and J. C. Jochims, *Angew. Chem. Int. Ed.*, 1966, **5**, 420-420.
10. J. C. Jochims and G. Karich, *Tetrahedron Lett.*, 1974, **15**, 4215-4218.
11. J. C. Jochims and G. Karich, *Tetrahedron Lett.*, 1976, **17**, 1395-1398.
12. F. W. Nader and A. Brecht, *Angew. Chem. Int. Ed.*, 1986, **25**, 93-94.
13. N. Suzuki, N. Ohara, K. Nishimura, Y. Sakaguchi, S. Nanbu, S. Fukui, H. Nagao and Y. Masuyama, *Organometallics*, 2011, **30**, 3544-3548.
14. J. A. Januszewski, D. Wendinger, C. D. Methfessel, F. Hampel and R. R. Tykwinski, *Angew. Chem. Int. Ed.*, 2013, **52**, 1817-1821.
15. E. W. Weisstein, Cylindrical Coordinates. From MathWorld--A Wolfram Web Resource., http://mathworld.wolfram.com/CylindricalCoordinates.html).
16. A. Jensen, M. H. Garner and G. C. Solomon, *ChemRxiv, Preprint*, 2019, DOI: 10.26434/chemrxiv.7851587.
